# Modulation of Mrp1 (ABCc1) and Pgp (ABCb1) by Bilirubin at the Blood-CSF and Blood-Brain Barriers in the Gunn Rat

**DOI:** 10.1371/journal.pone.0016165

**Published:** 2011-01-31

**Authors:** Silvia Gazzin, Andrea Lorena Berengeno, Nathalie Strazielle, Francesco Fazzari, Alan Raseni, J. Donald Ostrow, Richard Wennberg, Jean-François Ghersi-Egea, Claudio Tiribelli

**Affiliations:** 1 Centro Studi Fegato, Basovizza Trieste, Italy; 2 Brain-i at INSERM U842, Université de Lyon, Lyon1, Faculté de Médecine Laennec, Lyon, France; 3 S.C. Laboratorio Analisi Cliniche IRCCS Burlo Garofolo, Trieste, Italy; 4 Gastroenterology/Hepatology Division, University of Washington School of Medicine and VA Puget Sound Health Care System/Seattle Division, Seattle, Washington, United States of America; 5 Division of Neonatology, University of Washington School of Medicine, Seattle, Washington, United States of America; 6 INSERM U842, Université de Lyon, Lyon1, Faculté de Médecine Laennec, Lyon, France; Universidade Federal do Rio de Janeiro, Brazil

## Abstract

Accumulation of unconjugated bilirubin (UCB) in the brain causes bilirubin encephalopathy. Pgp (ABCb1) and Mrp1 (ABCc1), highly expressed in the blood-brain barrier (BBB) and blood-cerebrospinal fluid barrier (BCSFB) respectively, may modulate the accumulation of UCB in brain. We examined the effect of prolonged exposure to elevated concentrations of UCB on expression of the two transporters in homozygous, jaundiced (jj) Gunn rats compared to heterozygous, not jaundiced (Jj) littermates at different developmental stages (2, 9, 17 and 60 days after birth). BBB Pgp protein expression was low in both jj and Jj pups at 9 days (about 16–27% of adult values), despite the up-regulation in jj animals (2 and 1.3 fold higher than age matched Jj animals at P9 and P17–P60, respectively); Mrp1 protein expression was barely detectable. Conversely, at the BCSFB Mrp1 protein expression was rather high (60–70% of the adult values) in both jj and Jj at P2, but was markedly (50%) down-regulated in jj pups starting at P9, particularly in the 4^th^ ventricle choroid plexuses: Pgp was almost undetectable. The Mrp1 protein down regulation was accompanied by a modest up-regulation of mRNA, suggesting a translational rather than a transcriptional inhibition. *In vitro* exposure of choroid plexus epithelial cells obtained from normal rats to UCB, also resulted in a down-regulation of Mrp1 protein. These data suggest that down-regulation of Mrp1 protein at the BSCFB, resulting from a direct effect of UCB on epithelial cells, may impact the Mrp1-mediated neuroprotective functions of the blood-cerebrospinal fluid barrier and actually potentiate UCB neurotoxicity.

## Introduction

Severe jaundice from unconjugated bilirubin (UCB) can occur transiently in newborn infants with immature hepatic conjugating capacity [1], [2], [3], [4], [5] and lifelong in patients with Crigler-Najjar type I disease [6], [7]. Over 99.9% of UCB in blood is bound to plasma proteins (primarily albumin) that do not enter the brain [8], [9], [10]. Only the small fraction of unbound bilirubin may diffuse into the brain and cerebrospinal fluid (CSF). With severe jaundice, the serum binding sites approach saturation and unbound UCB (free bilirubin, Bf) will rise dramatically even at lower biliruibin/albumin molar ratios [2], [11]. Under these conditions, the accumulation of UCB in brain (kernicterus) can produce toxicity and result in permanent brain injury.

The exchange of unbound UCB between the blood and the brain may be modulated by two blood-brain interfaces that control cerebral homeostasis [12]. Directly contacting the neuroglia, the blood-brain barrier consists of tightly bound, specialized endothelial cells lining the brain microvessels [13]. A second interface, the blood-cerebrospinal fluid barrier is provided by the epithelial cells of the choroid plexuses, and controls exchanges between plasma and CSF. Both barriers display a large surface area for exchanges [14]. Tight junctions [15], [16] preclude the paracellular passage of hydrophilic compounds [17], while transporters [18] and metabolizing enzymes [19], [20], [21], [22] control the neural access of lipid-soluble substrates.

Two trans-membrane proteins, belonging to the ATP binding cassette (ABC) family, have been identified as potential UCB transporters, which export the pigment from the cells. MDR1 (ABCb1: Pgp, Mdr1a/1b in rodents) displays a low affinity for UCB [23] and MRP1 (ABCc1: Mrp1 in rodents) that possess a very high affinity for UCB (Km for Bf = 10 nM) [24]. In rat as well as in human brain, Pgp is specifically expressed in microvessels, localized at the luminal (blood) side of the endothelium, while Mrp1 is mostly localized at the basolateral membrane of the choroidal epithelium, facing the stromal/blood space. Pgp protein increases during post-natal development, whereas Mrp1 is highly expressed in choroid plexuses, even at birth [14], [18], [25], [26]. Both ABC transporters may participate in limiting the entry of UCB by increasing its export from the central nervous system [27], [28].

In this study we investigated the effect of sustained unconjugated hyperbilirubinemia on the developmental protein expression of Mrp1 in the lateral and 4^th^ ventricle choroid plexuses, and of Pgp in brain microvessels. We used immature and adult Gunn rats [29], a well established animal model for chronic unconjugated hyperbilirubinemia and kernicterus [30]. Homozygous, recessive (jj) Gunn rats, like patients with Crigler-Najjar syndrome type I (CNS I), develop severe, lifelong, non-hemolytic, unconjugated hyperbilirubinemia, due to the congenital absence of UDP- glucuronosyl transferase (UDPGT: EC 2.4.1.17) 1A1, the enzyme that synthesizes excretable conjugates of UCB. The same enzyme has transient low activity in jaundiced human newborns [31], [32]. Heterozygous Jj animals, used as controls, have only a minimally reduced activity of UGT1A1 compared with Sprague-Dawley animals [33].

## Materials and Methods

### Animals

Gunn rats (Hds Blue:Gunn-UDPGTj) [29], originally purchased from Harlan (Harlan, IL, USA) in 2006, were maintained in the animal facility of the University of Trieste. Animal care and procedures were conducted according to the guidelines approved by Italian Law (decree 116-92) and by European Community directive 86-609-EEC. Sacrifice was performed after anesthesia (isofluorane) in order to avoid animal pain or stress, within the law 116-92. Because animals spontaneously develop the pathology, and no treatments have been applied, additional ethical approval was not required. All efforts were made to avoid suffering of animals.

Littermates were obtained by breeding Jj females with jj males. Parturitions were synchronized to obtain a sufficient number of littermate pups of each genotype and post-natal age (P±24 hrs). Jj pups from the same litter were used as controls for jj animals. Post-natal ages to study were selected based on developmental vulnerability to bilirubin neurotoxicity established by others [34], [35], [36], [37], [38]. The number of rats pooled for each batch and the number of batches analyzed for each transporter are listed in Table 1.

**Table 1:** Animals and batches of samples used for the ABC transporter analysis.

Post-natal age		P2	P9	P17	P60	P120
**MVs**	**Animals** *	N.P.	8	6	4	N.P.
	**Batches**	N.P.	3	4	5	N.P.
**CPs**	**Animals** *	4	4	3	4	3
	**Batches**	4	4	4	4	3

MVs: microvessels; CPs: choroid plexuses;

*: Animals pooled together to generate one batch. N.P.: not performed.

### Plasma bilirubin determination

A heparinized sample of blood was obtained by jugular puncture from each anesthetized animal. After centrifugation (1500 g for 20 min at room temperature), plasma was collected under dim light to minimize bilirubin photo-oxidation and immediately frozen at −20°C until assayed. The diazo-reaction [39] (Boehringer-Mannheim Kit 1552414, Monza, Italy) was used to quantify total plasma bilirubin. Hemolyzed samples were discarded. More than 12 animals were used at every post-natal age for each genotype.

### Tissue dissection and preparation

Animals were euthanized under isofluorane anesthesia by decapitation (within the law 116-92). Two lateral ventricle choroid plexuses, one from each hemisphere, 4^th^ ventricle choroid plexuses, cerebral cortices and cerebella were dissected individually from four P2, P9, P17 and P60 animals of each phenotype (see Table 1) in Krebs-Ringer buffer (in mM: 135 NaCl, 4 KCl, 2.2 CaCl_2_, 1.2 MgCl_2_, 6 NaHCO_3_, 10 HEPES, 5 glucose, pH 7.4), at 4°C under stereomicroscope vision. Spleens from Jj and jj animals were also dissected for Mrp1 analysis. Brain microvessels were obtained as described previously [18]. Briefly, pooled cerebral cortices were cleaned from all apparent meninges under a stereomicroscope. For mature animals (P60), tissues were homogenized in a Dounce-type glass-glass homogenizer after the addition of 5 vol/g tissue of 1% bovine serum albumin-supplemented Krebs-Ringer. The microvessels were separated from larger vessels and brain parenchyma material by filtering through decreasing pore diameter mesh sieves and the myelin was removed by centrifugation on a 17.5% 70 KDa-Dextran gradient in Krebs-Ringer buffer at 3000 g for 30 min. The microvessel fraction retained on the 40 µm sieve was recovered in 0.1% albumin in Krebbs-Ringer buffer, centrifuged, and suspended in a small volume of the same buffer. Because of the fragility of brain tissue, cerebral cortices of P17 animals were homogenized by eight loose-type pestle strokes, while five loose-type pestle strokes were used for P9 pups. Preparations contaminated with larger vessels or tissue remnants were discarded. All steps were performed at 4°C within 4 hours of decapitation. Samples were stored at −80°C until use for Western blot.

### Sample preparation and Western blot quantification

Freshly isolated microvessels, and lateral and 4^th^ ventricle choroid plexuses were homogenized in buffer (0.25 M Sucrose; 50 mM K Phosphate; 1 mM EDTA; 0.1 mM DTT; pH 7.4), using a Dounce-type glass-glass homogenizer. Total protein content was determined using bicinchoninic acid (BCA), following the manufacturer's protocol (Procedure # TPRO 562, Sigma, St Louis, MO, USA).

Proteins were separated on 10% SDS-polyacrylamide gels. Before transfer, the gel was cut longitudinally; the lower part (containing the actins) was blotted on PVDF membrane (0.2 µm; BioRad Laboratories, Hercules, CA, USA) at 100 V in transfer buffer (25 mM Tris-Base; 192 M glycine, 0.1% SDS; 20% methanol) for 1h, while the upper part of the gel (Mrp1 and Pgp) was blotted for 2 hr, to achieve maximal transfer. Completeness of transfer was assessed by lack of Coomassie blue coloration of the gels after blotting.

**Figure 1:**
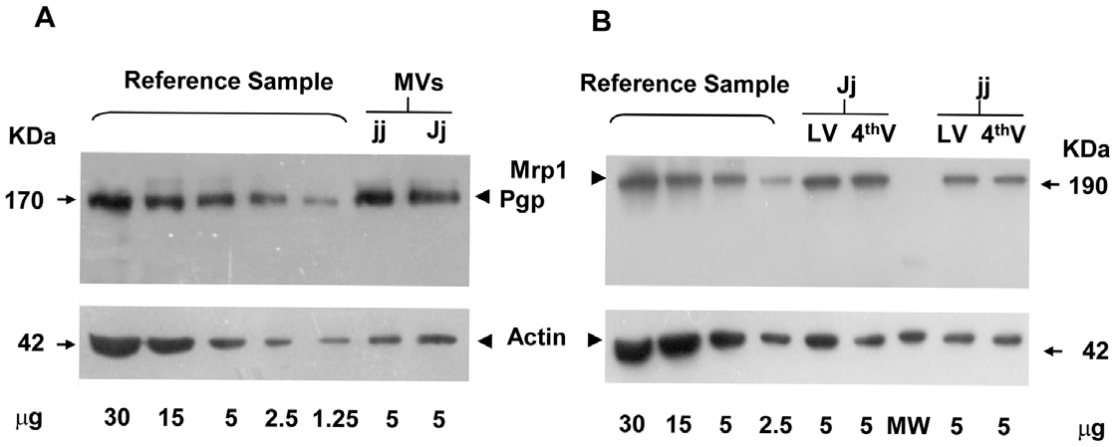
Representative Western blot for Pgp, Mrp1 and Actin on Gunn preparations. Pgp expression was analyzed on microvessels (MVs) preparations (A, upper panel). Mrp1 was quantified in lateral and 4^th^ ventricle (LV and 4^th^V) choroid plexuses (B, upper panel). Actin staining (both A and B, lower panel) was used to normalize the amount of samples loaded. For the quantification procedure, see the material and methods section.

PVDF membranes were saturated in blocking solution (4% non fat milk in 20 mM Tris base; 500 mM NaCl, pH 7.4, supplemented with 0.2% Tween 20) for 1 hr, incubated overnight at 4°C with antibodies against either Mrp1 (A23, produced in our laboratory and commercialized by Alexis, Lausanne, Switzerland) [40], Pgp (Abcam PLC, Cambridge, UK), or actin (Sigma; St Louis, MO, USA). Antibodies were added at a final concentration of 1.0, 4.8 and 1.2 µg/mL, respectively. Membranes were then incubated for 2 hr with horseradish peroxidase-conjugated secondary antibodies (both anti-mouse and anti-rabbit at 0.8 µg/mL; Sigma; St Louis, MO, USA) in blocking solution. Specificity of the antibodies against the two ABC transporters in rats was previously confirmed [18], and checked again in this work (Fig. 1).

Horseradish peroxidase activity was developed using a chemiluminescence procedure (Millipore, Billerica, MA, USA), according to the manufacturer's protocol, and visualized on X-ray films (BioMax Light, Kodak Rochester, NY, USA). The optical density profile of each band revealed on the X-ray film was quantified by ImageQuant software (GE Healthcare Europe GmbH, France).

Reference controls to quantify the relative contents of Pgp and Mrp1 were obtained by serial dilutions of pooled microvessels and 4^th^ ventricle choroid plexuses obtained from P60 Jj Gunn rats and run for each Western blot procedure (Fig. 1). A standard curve was generated by non-linear regression (MMF model, each r>0.99) of plots of optical density values versus the amount of reference sample protein. Relative expression of the ABC transporters, normalized for the actin signal, was calculated by CurveExpert 1.38 software (Hixon, TN, USA). The results are expressed as mean ± S.D., n = 3 to 5 sample batches for each post-natal age (see Table 1).

### Immunohistochemical detection of Mrp1 in rat brain sections

Several rat brains of each genotype and developmental stage were immediately snap-frozen after decapitation, and 10 µm-thick slices were cryo-sectioned, using a Micron HM550 cryostat (Bio-Optica, Milan, Italy). Sections were fixed at room temperature in 4% paraformaldehyde in phosphate buffer (PBS; in mM: NaCl 150; Na_2_HPO_4_ 12; KH_2_PO_4_ 2; pH 7.4) for 10 min, then blocked for 1 hr in 5% BSA, 5% Normal Goat Serum, 0.3% Triton in PBS. After overnight incubation at 4°C with the anti-Mrp1 antibody (final concentration 1 µg/mL) in blocking solution, secondary anti-rabbit Alexa-conjugated antibodies (MolecularProbes, Carlsbad, CA, USA, 2 µg/mL, in 0.3% Triton-PBS) were added and incubated for 2 hr at room temperature.

Nuclei were stained with Hoechst 33521 (0.1 µg/mL) in PBS for 10 min at room temperature and sections were mounted with cytomation fluorescence mounting medium (S3023, Dako, Glostrup, Denmark). Negative controls were performed by omitting the first antibody. Fluorescent immunoreactions were observed using a Leica DM200 fluorescence microscope, equipped with the Digital Camera Leica DFC490 and the software Leica LAS Image Overlay (Leica, Ernst-Leitz-Strasse 17–37, Wetzlar, D-35578, Germany).

### Real Time-PCR analysis of Mrp1 mRNA levels in choroid plexuses of P9 animals

Lateral and 4^th^ ventricle choroid plexuses of Jj and jj Gunn rats were collected in TriReagent (Sigma-Aldrich, St Louis, MO, USA). Total RNA was isolated according to the manufacturer's instructions. Briefly, tissues were lysed with the reagent, chloroform was added and cellular RNA was precipitated by isopropyl alcohol. After washing with 75% ethanol, the RNA pellet was dissolved in nuclease-free water and stored at −80°C until further analysis. RNA was quantified spectrophotometrically at 260 nm, and the RNA purity was evaluated by measuring the ratio A_260_/A_280_, considering RNAs with appropriate purity those showing values between 1.8 and 2.0. RNA integrity was evaluated by gel electrophoresis.

Total RNA (1 µg) in 15 µl of nuclease-free water was added to 4 µL reaction mix, and 1 µL reverse transcriptase using the iScript™cDNA Synthesis kit (Bio-Rad Laboratories, Hercules, CA, USA) according to the manufacturer's instructions. The reaction was performed for 5 min at 25°C (annealing), 30 min at 42°C (cDNA synthesis), and 5 min at 85°C (enzyme denaturation).

Three house-keeping genes (beta-actin, 18S and GAPDH), each corresponding to a different function in the cell, were employed to normalize the results. The PCR reaction was performed on 25 ng cDNA with gene-specific sense and anti-sense primers for Mrp1, β-actin, GAPDH (all Mrp1; β-actin and GAPDH 250 nM) and 18S (100 nM) (Table 2) with iQ SYBR Green Supermix (Bio-Rad Laboratories, Hercules, CA, USA) in an i-Cycler IQ thermocycler (Bio-Rad Laboratories, Hercules, CA, USA). The thermal cycler conditions consisted of 3 min at 95° and 40 cycles each at 95°C for 20 s, 60°C for 20 s and 72°C for 30 s. Specificity of the amplification was verified by a melting-curve analysis, performed immediately after the amplification protocol, under the following conditions: 1 min denaturation at 95°C, 1 min annealing at 55°C, and 80 cycles of 0.5°C increments (10 s each) beginning at 55°C. Non-specific products of PCR were not found in any case. A standard curve was generated using a “calibrator” cDNA (chosen among the cDNA samples), which was serially diluted and analyzed for all genes to determine the amplification efficiencies of individual genes. The relative quantification was made using Genex software (Bio-Rad Laboratories, Hercules, CA, USA) based on ΔΔCt method, taking into account the efficiencies of individual genes and normalizing the results to the three house-keeping genes. The levels of Mrp1 mRNA were expressed relative to a selected 4^th^ ventricle choroid plexuses Jj reference sample. The results are expressed as mean ± S.D., n = 3 pools of choroid plexuses dissected from 4 animals.

**Table 2:** Primers used for transcript analysis, amplicon length and amplification efficiency.

Gene	Accession Number	Sense	Antisense	Amplicon Lenght (bp)	Efficency (%)
**Mrp1**	NM_022281	ATGGTGTCAGTGGTTTAGG	TGTGGGAAGAAGAGTTGC	111	99.2
**B-Actin**	NM_031144	ATCGGCAATGAGCGGTTCC	AGCACTGTGTTGGCATAGAGG	149	81.5
**GAPDH**	NM_017008	CCATCACCATCTTCCAGGAG	CCTGCTTCACCACCTTCTTG	576	88.1
**18S**	X01117	TAACCCGTTGAACCCCATT	CCATCCAATCGGTAGTAGCG	150	74.7

### Effect of bilirubin on Mrp1 protein in an *in vitro* model of the blood-CSF barrier

Choroid plexus epithelial cells were isolated and cultured on Costar Transwell Clear inserts (0.4 µm pore size, 6.5 mm diameter) as previously described [41], [42]. Cells were treated in the basolateral compartment of the bicameral system and exposed for 6 consecutive days to daily renewed 7.5 µM or 20 µM UCB, starting on day 4 after plating. UCB was dissolved in DMSO (0.16 and 0.4% final volume, for low and high dose, respectively) and diluted in culture medium supplemented with 11.5% fetal bovine serum containing 30 µM albumin. UCB concentration was adjusted to yield bilirubin/albumin ratios calculated to have free bilirubin concentrations of approximately 40 and 140 nM [43]. Control monolayers were treated in parallel with the same concentration of DMSO. On day 10, filters were cut and transferred into 1× Lysis Buffer (Cell Signalling Technology, Saint-Quentin Yvelines, France) and kept at −80°C, until processed for Western blot analysis of both Mrp1 and actin as previously described in detail [18]. Duplicate monolayers were included for each condition. Actin was used to assess protein load of each sample.

### Statistical analysis

Data are reported as mean ± SD. Statistical differences between hyperbilirubinemic animals and controls (Jj) were analyzed using paired Student's t-test for Western blot ABC transporter quantification. Anova following by a Tukey-Kramer test was used to assess variation in total plasma bilirubin concentration over time. One tailed Student'*s* test for unequal variances was applied to assess differences in cerebellar growth and plasma bilirubin concentration and in mRNA levels between Jj and jj animals. Differences were considered statistically significant at a p value lower than 0.05.

## Results

### Plasma bilirubin and cerebellar growth

**Figure 2:**
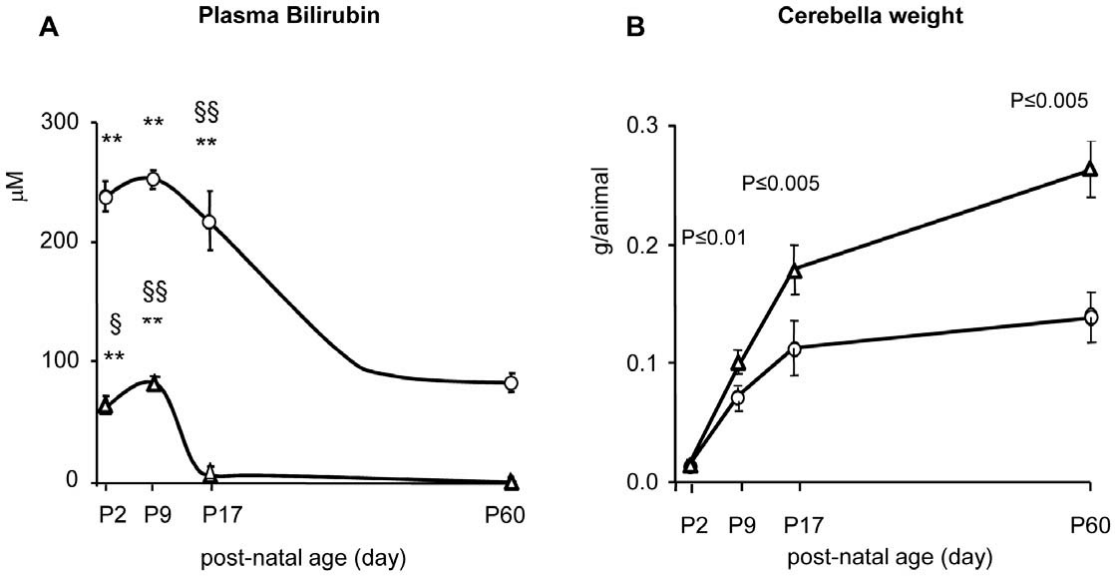
Plasma bilirubin and cerebellar weight in jj and Jj Gunn rats. The values are expressed as mean ± SD of 12–20 animals for each age and genotype. Round filled dots: jj animals; empty triangles: Jj controls. Panel A: total plasma bilirubin (Tbil) amount in developing hyperbilirubinemic (jj) and control (Jj) Gunn rats. Tbil concentration differences between jj and age-matched Jj animals were all statistically significant (P≤0.005). Within each phenotype: **: p<0.005 vs. P60; § and §§ : p<0.05 and 0.005 respectively *vs.* next age group. Panel B shows the cerebellar growth expressed as g/animal. The level of significance is indicated by comparison with age-matched not jaundiced Jj animals.

A transient postnatal increase in total plasma bilirubin concentration was observed in Jj animals, decreasing to the low adult values by P17 (Fig. 2A). The total bilirubin in plasma of jj rats was significantly higher (p<0.005) than in Jj rats at all post-natal ages and reached the peak level (250 µM) at P9. A hallmark of bilirubin toxicity in jj rats is cerebellar hypoplasia [37], [44], [45]. Cerebellar weights in jj and Jj animals were identical at P2, but cerebellar growth was impaired thereafter in jj animals and almost completely arrested by P17 (Fig. 2B). Thus, the jj cerebellar weight was 25% lower at P9 (p<0.01), 40% lower at P17 (p<0.005) and 50% lower at P60 (p<0.005) than in Jj animals.

### Characterization of isolated microvessels and choroid plexus tissues

**Figure 3:**
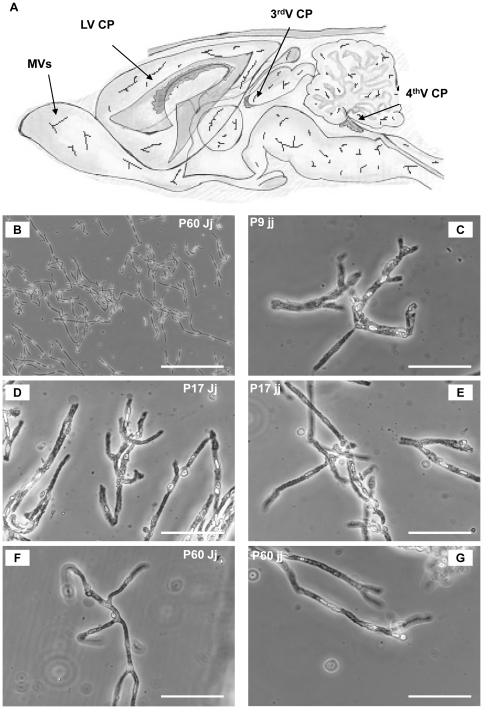
Blood brain barrier localization in rat brain, and microvessels isolated from rats. Cartoon representation of the lateral, 3^rd^, 4^th^ ventricle choroid plexuses (LV CP, 3^rd^ V CP and 4^th^V CP, respectively) and microvessels (MVs) localization in rat brain (A). Representative microvessels freshly isolated from rat brains. Freshly isolate MVs in 0.1% albumin in Krebbs-Ringer buffer were placed on a microscope slide and directly observed by phase contrast microscopy: In B scale bar 100 µm; in C, D, E, F and G scale bar 25 µm. P: post-natal age in days. jj: hyperbilirubinemic rats; Jj: controls.

It was technically impossible to obtain microvessel preparations of adequate purity from rat brain tissue at ages earlier than 9 days [18]. On phase contrast microscopic examination, microvessels preparations consisted mostly of long, branched capillaries, with the thin structure of the microvessels better appreciated at higher magnifications (Fig. 3, B–G). No contaminating larger vessels, cellular debris, or meninges were observed (Fig. 3, B), and red blood cells were rarely found. Despite a softer consistency of the cortices dissected from jaundiced jj rats, vascular morphology was similar to Jj rats and no cellular alteration (granulations, bubbles, etc.) was detected (Fig. 3, panel D, F for Jj and panel C, E, G for jj). Similarly, no difference was noted in the dimensions or morphology of either lateral or 4^th^ ventricle choroid plexuses dissected from jaundiced or control Gunn rats.

### Pgp protein expression in microvessels

**Figure 4:**
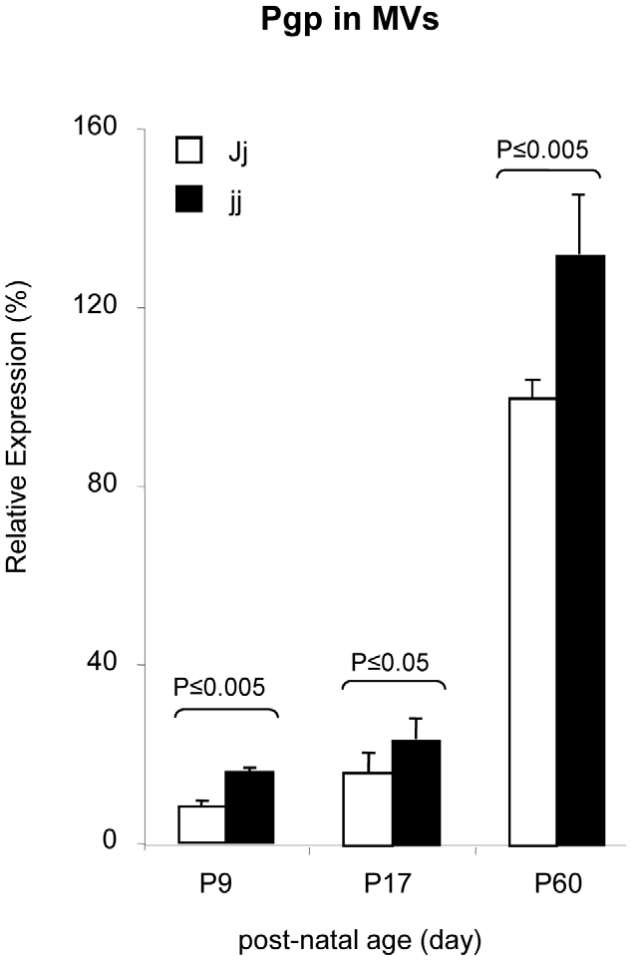
Pgp relative expression in isolated Jj and jj Gunn rat microvessels. The Pgp amount in each sample is expressed as % of the Pgp amount in the Reference Sample (Pooled P60 Jj MVs preparations). White bars: heterozygous (Jj) rats, black bars: hyperbilirubinemic homozygous (jj) animals. The values are expressed as mean ± SD. Statistical significance is indicated.

In agreement with previous data on rats and mice, Pgp expression in microvessels strongly increased from the early post natal age to the adult age [18], [46], [47]. Pgp concentration was higher in jj animals than in Jj littermates at every post-natal age analyzed (Fig. 4). The maximal difference was observed at P9, when the Pgp amount in jj was 1.8-fold the level expressed by Jj age-matched controls (p<0.01). Differences were smaller at later developmental ages (1.4-fold at P17, and 1.3-fold at P60), but remained statistically significant (p<0.05). Notwithstanding bilirubin-induced up-regulation, the Pgp concentration in 9 day old jj rats was still only 16% of the level found in mature Jj rats.

### Mrp1 protein and mRNA expression in choroid plexuses

**Figure 5:**
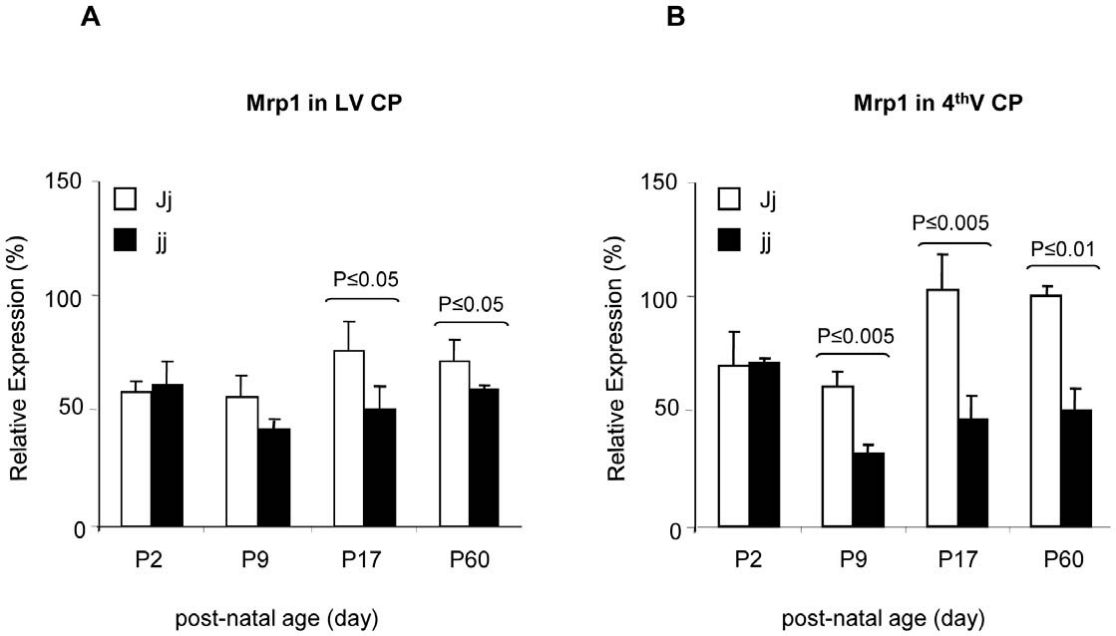
Mrp1 relative expression in the lateral and 4^th^ ventricle choroid plexuses dissected from Jj and jj Gunn rats. The Mrp1 protein amount present in each sample is expressed as % of the Mrp1 amount in the Reference Sample (pooled P60 Jj 4^th^V CP preparations). White bars: heterozygous (Jj) rats, black bars: hyperbilirubinemic homozygous (jj) animals. 4^th^V CP: Forth Ventricle Choroid Plexuses; LV CP: Lateral Ventricles Choroid Plexuses. Statistical significance is indicated.

The expression of Mrp1 in the choroid plexuses was high at birth, with values similar to adult levels (Fig 5, A and B). Mrp1 expression in jj and Jj rats were comparable at P2, but Mrp1 expression in jj animals fell to a nadir at P9 and remained statistically lower than in Jj animals at later ages. At P60, the Mrp1 level of the jj rats was 50% lower in the 4^th^ ventricle choroid plexuses (p<0.001) and 30% lower in the lateral ventricle choroid plexuses (p<0.05) (Fig 5, B and A, respectively), than in Jj animals.

**Figure 6:**
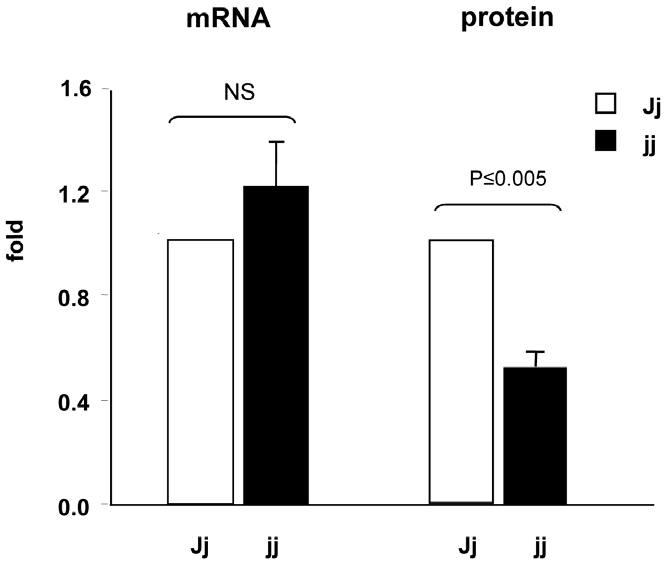
Comparison of Mrp1 mRNA and protein relative expression in choroid plexuses. White bars: heterozygous (Jj) P9 rats, black bars: hyperbilirubinemic homozygous (jj) P9 animals. The values are expressed as mean ± SD. Statistical significance is reported. NS: not significant difference.

To assess the specificity of the decrease in Mrp1 found in CPs, we analyzed the expression of this transporter in the spleen, an organ that highly expresses Mrp1. In contrast to choroid plexuses, Mrp1 expression in the spleen did not differ between jj and Jj rats at any age (P9: 100.8±2.6 vs. 101.8±2.5; P17: 101.2±9.0 vs. 90.4±8.1, Jj vs. jj animals). When Mrp1 mRNA was analyzed by real time PCR in P9 choroid plexus preparations, the expression was comparable between Jj and jj rats, despite a 50% drop in Mrp1 protein (Fig. 6).

The absence of either molecular weight shift or lower molecular weight bands for Pgp in micro vessels and Mrp1 in choroid plexuses in jj preparations suggests that hyperbilirubinemia does not alter the post-translational maturation nor produce degradation of these transporters. In both JJ and jj Gunn rats, Pgp expression in choroid plexuses and Mrp1 expression in microvessels were barely detectable (data not shown) as previously shown in Sprague-Dawley rats [18].

### Immunofluorescence analysis of Mrp1 cellular localization in rat brain

**Figure 7:**
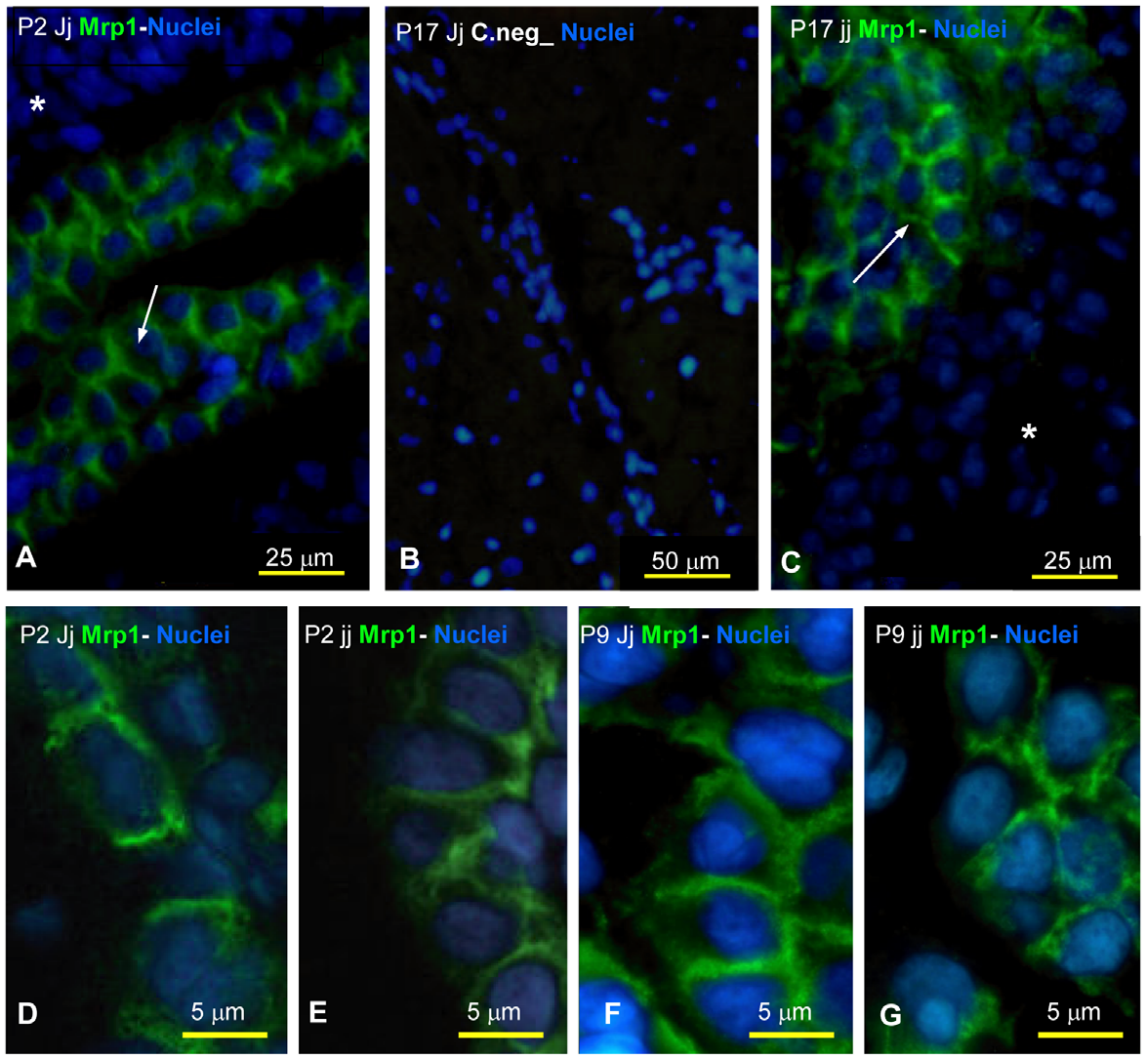
Immunofluorescence for Mrp1 on Gunn rat brain slices during post-natal development. A, B, D, F: heterozygous (Jj) animals. C, E, G: hyperbilirubinemic homozygous (jj) animals. A negative control obtained omitting the first antibody is shown in B. A and C, show the specific expression of Mrp1 at the blood-cerebrospinal fluid barrier of Jj and jj Gunn rats. Green: Mrp1;Blue: Nuclei; → : basolateral side; * : parenchyma. In D, E (P2 Jj vs jj) and F,G (P9 Jj vs jj) magnifications showing Mrp1 localization at the basolateral side of choroidai plexus cells.

Mrp1 was localized at the basolateral membrane of choroid plexus epithelial cells, in both Jj and jj Gunn rat brain slices, even at P2 (Fig. 7, A, D and E), in agreement with previous studies in Sprague-Dawley rats [18]. No cytoplasmic staining was detected in jj rats (compare D with E, and F with G), suggesting that UCB is unable to reallocate the transporter *in vivo*. When the nuclei were counterstained by Hoechst 33422, no apoptotic bodies were detected, in keeping with the absence of macroscopic cellular damage due to UCB.

### Effect of UCB on Mrp1 protein expression in cultured choroidal epithelium

**Figure 8:**
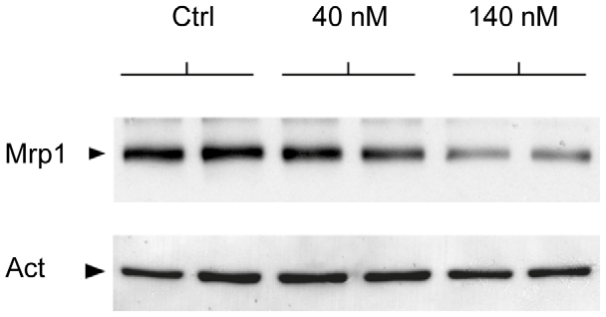
Western blot analysis of Mrp1 protein in the choroidal epithelium following bilirubin treatment. Duplicate monolayers of choroidal epithelial cells were included for each experimental condition. Following daily basolateral treatment with 40 or 140 nM unbound bilirubin for 6 consecutive days, filters were treated in exactly the same conditions. The homogeneity in protein loading is reflected by the actin band revealed on the lower part of the membrane.

Monolayers of choroidal epithelial cells were cultured in bicameral devices, reconstituting *in vitro* the barrier properties of the blood-CSF interface. Cells were exposed to 2 different concentrations of unbound bilirubin (40 and 140 nM, which mimic levels measured in physiological and pathological jaundice, respectively) at the basolateral membrane site for 6 consecutive days to mimic the *in vivo* prolonged postnatal exposure. This treatment resulted in a decrease in Mrp1 protein at the higher bilirubin concentration (Fig. 8). This effect was not accompanied by any alteration in the paracellular permeability of the monolayers to sucrose (data not shown), and therefore does not reflect a pleiotropic toxicity. These data are in agreement with our *in vivo* results and suggest that the reduction of Mrp1 protein observed in jj animals in the blood-CSF barrier results from a direct action of UCB on barrier cells.

## Discussion

UCB can enter brain parenchyma either directly *via* the blood-brain barrier, or by transfer across the choroid plexuses. The ABC transporters, Pgp and Mrp1, have been proposed to modulate brain entry of UCB [14]. *In vitro*, Pgp, mainly expressed in microvessels, is a UCB transporter weaker than Mrp1 [23], [24], the latter being dominant in the choroid plexuses [18]. The effects of prolonged exposure to high UCB levels *in vivo* as seen in severely jaundiced newborns or Crigler-Najjar type I patients, on the post natal-expression of Pgp and Mrp1 at the blood-brain interfaces have not been reported.

We observed an up-regulation of Pgp protein in microvessels, and a down-regulation of Mrp1 in choroid plexuses isolated from hyperbilirubinemic jj Gunn rat pups as compared with their non-jaundiced Jj littermate controls. Mechanisms mediating these opposite responses are still uncertain. UCB has been show to affect the cellular redox [48], [49], [50], [51] state and Pgp expression is increased by oxidative stress [52], [53], suggesting that oxidative stress may be a mediator of UCB-induced Pgp protein level at the blood-brain barrier.

UDPGT activity is present in rat choroid plexuses [19], [54] and glucuronosyl conjugates are one of the substrates for Mrp1. In jj Gunn rats, UGT1A1 activity is completely abolished by a genetic mutation, suggesting that the resulting limited availability of Mrp1 substrates might lead to an Mrp1 down regulation in jj animals. However, the normal protein expression of Mrp1 in choroid plexuses of P2 pups (Fig 5) and in spleen and in liver (data not shown) indicates that the Mrp1 protein reduction is not due to the decrease in UGT activity but rather to a direct effect of the prolonged exposure to high levels of UCB. This is further supported by the *in vitro* data showing that UCB is *per se* able to reduce the protein content of Mrp1 in choroid plexuses.

The lack of apparent degradation products of Mrp1 (Fig. 1, Western blot), the normal localization at the basolateral membrane (Fig. 7, immunofluorescence), together with the comparable levels of mRNA in hyperbilirubinemic and control animals (Fig. 6) suggest that the reduction in the content of Mrp1 protein observed in jj rats occurs at the translational level. A similar effect, i.e. reduced protein without changes in the mRNA level or modifications in sub-cellular localization was observed for Mrp2 and Bile Salt Export Pump (BSEP; ABCB11) following lipopolysaccharide exposure in human liver [55], [56].

Lipophilic compounds such as UCB cross cellular membranes mainly by passive diffusion [17], [57]. The protein up-regulation of Pgp we observed is consistent with a defensive role proposed for this transporter against bilirubin entry into brain. However, despite this up-regulation, the expression of Pgp in response to the elevated levels of UCB in jj Gunn rats in the first 2.5 weeks after birth (P2–P17) remained very low compared to adult values (16 and 27% of adult Jj level, respectively). This suggests that Pgp offers, at best, a marginal defense against bilirubin entry into brain during the critical period (P2–P17) when the UCB-related neurological damage occurs, as the diffuse yellow coloration of the brains demonstrates (not shown). At the same time, the Mrp1 protein level in the choroid plexuses of jj animals is significantly reduced, which may further impair the ability of the brain to protect itself from UCB accumulation and toxicity. This has been shown *in vitro* where silencing of Mrp1, but not Pgp, is associated with a greater cellular toxicity induced by UCB exposure [58].

Mrp1 expression in choroid plexuses matures early during development and is involved in regulating cerebral availability of endogenous biologically active compounds (leukotriene [59], steroid hormones [60], glutathione, glucuronide and sulfate conjugates [61], reduced glutathione [62]) and therapeutic drugs such as etoposide [63], [64]. In line with the functional relevance of these transport activities, the involvement of the transporter in inflammation [65] and oxidative stress [66] has been demonstrated *in vivo* in Mrp1 KO mice.

We speculate that Mrp1 protein down-regulation observed in jaundiced jj Gunn rat pups and maintained into adulthood, may increase the central nervous system accumulation of toxins or drugs, impair immune and endocrine response, and lead to increased oxidative stress impairing neuronal development independent of bilirubin itself. In summary, we provide evidence that major alterations in Pgp and Mrp1 expression are induced in the BBB and BCSFB, respectively, by prolonged exposure to elevated levels of UCB, as seen in jj Gunn rats.

Although the mechanisms of these alterations in expression are currently undetermined, we propose that the modulation of UCB transporters *in vivo* results from the direct exposure of barrier cells to bilirubin, possibly mediated in part by an alteration in the cellular redox state.
